# Supplementary material for: Effect of Individual Rate of Inbreeding, Recent and Ancestral Inbreeding on Wool Quality, Muscling Conformation and Exterior in German Sheep Breeds
Source: Animals (Basel). 2023 Oct 26;13(21):3329. doi: 10.3390/ani13213329 (PMC10648841; doi:10.3390/ani13213329)
Supplement: Supplementary file 1 [file animals-13-03329-s001.zip › Figure S1-S3.Heritabilities_Plots.pdf]

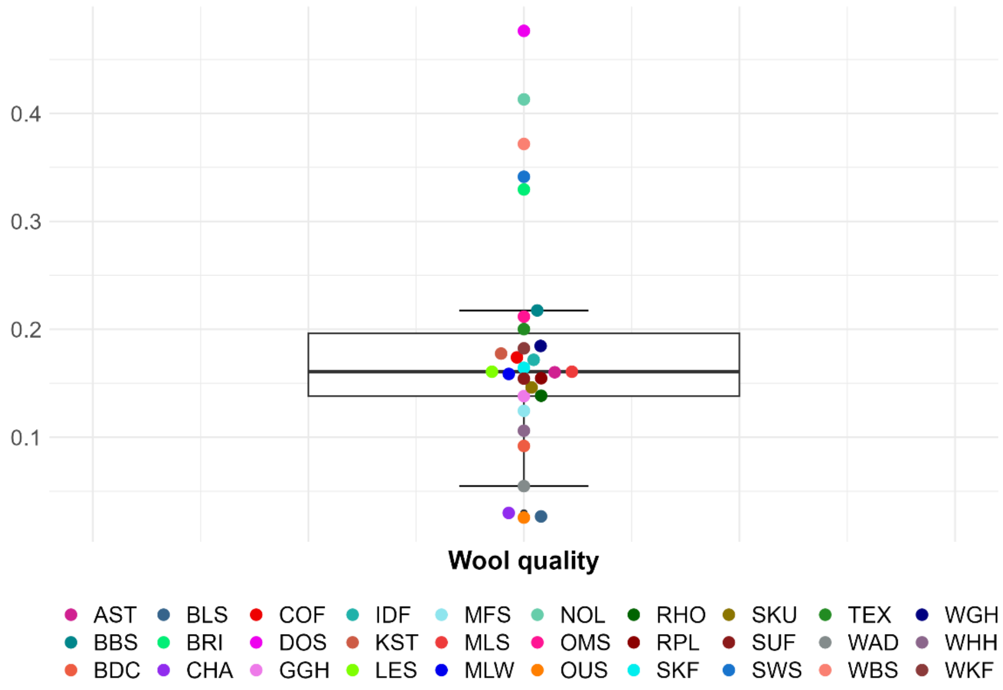

Figure S1. Estimated heritabilities for the trait of wool quality of 30 sheep breeds.

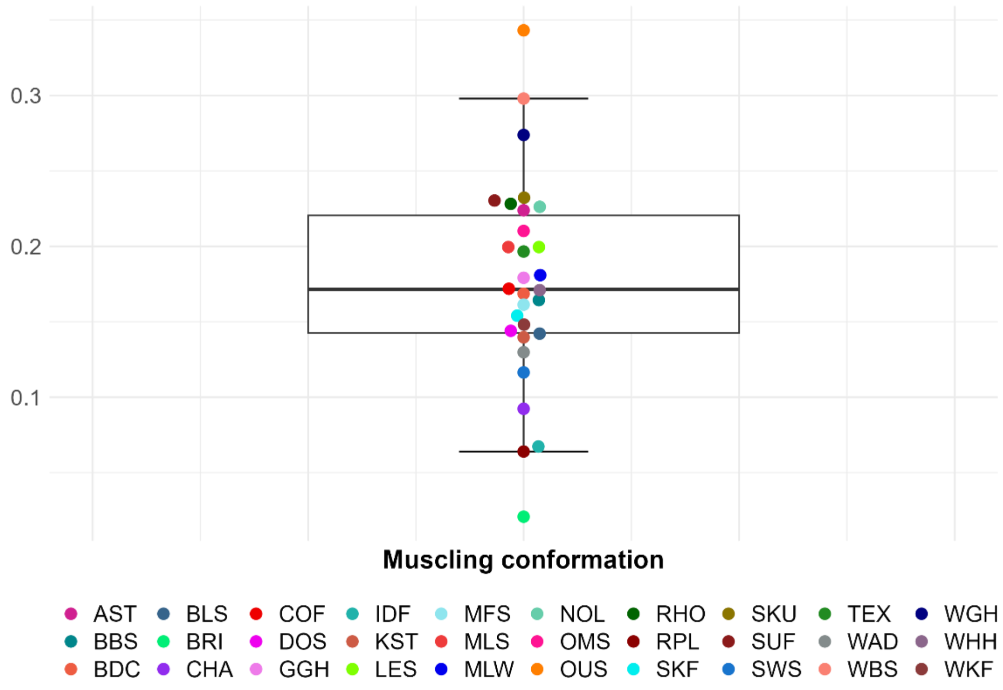

Figure S2. Estimated heritabilities for the trait of muscle conformation of 30 sheep breeds.

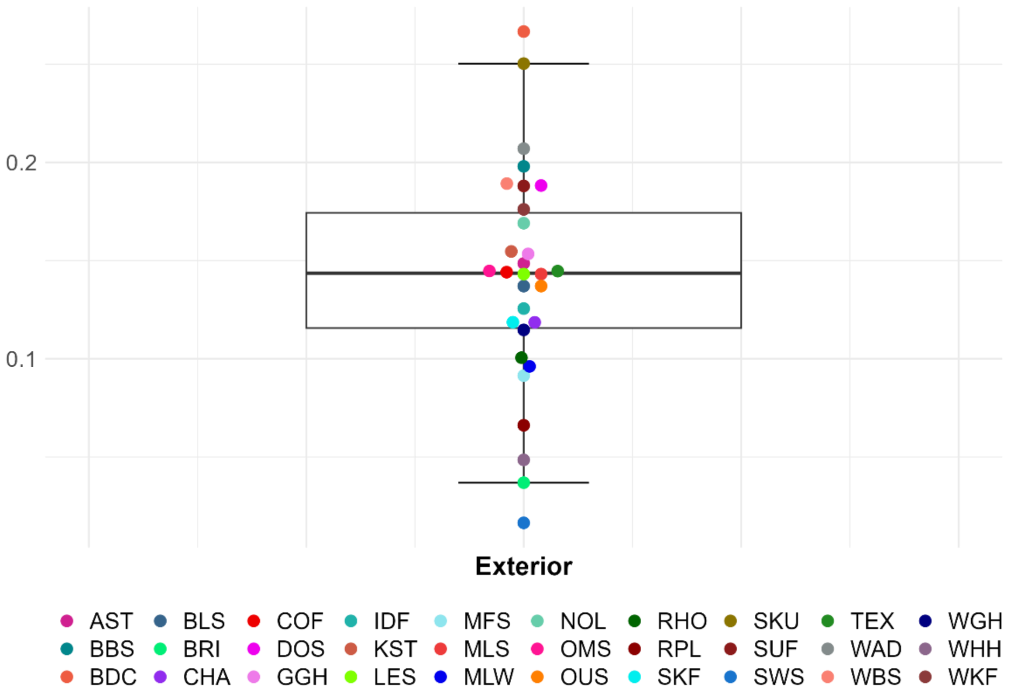

Figure S3. Estimated heritabilities for the trait of exterior of 30 sheep breeds.
