# Supplementary material for: Effect of Individual Rate of Inbreeding, Recent and Ancestral Inbreeding on Wool Quality, Muscling Conformation and Exterior in German Sheep Breeds
Source: Animals (Basel). 2023 Oct 26;13(21):3329. doi: 10.3390/ani13213329 (PMC10648841; doi:10.3390/ani13213329)
Supplement: Supplementary file 1 [file animals-13-03329-s001.zip › Table S3.Test criteria for hair sheep.pdf]

Table S3. Test criteria for the Barbados Blackbelly, Brown Hairsheep, Dorper and Nolana breeds.

Grade	Appearance in the summer months	Shearing
**9**	no wool or protruding hair	not required
**8**	1 wool or hair island (maximum diameter 10 cm)	
**7**	up to 3 wool or hair islands (maximum diameter 10 cm)	
**6**	Back covered up to 25 % with wool	
**5**	Back covered with more than 25 % wool	required
**4**	Back covered with wool 50% and more	
**3**	Back and flanks covered with wool	
**2**	loose wool fleece	
**1**	dense wool fleece	

The assessment of shedding behaviour in hair sheep according to this scheme should be done after the first wintering of the animals in the period June to October.
