# Supplementary material for: Effect of Individual Rate of Inbreeding, Recent and Ancestral Inbreeding on Wool Quality, Muscling Conformation and Exterior in German Sheep Breeds
Source: Animals (Basel). 2023 Oct 26;13(21):3329. doi: 10.3390/ani13213329 (PMC10648841; doi:10.3390/ani13213329)
Supplement: Supplementary file 1 [file animals-13-03329-s001.zip › Table S4.Breeding directions and endangered breeds.pdf]

Table S4. Breeding directions of German sheep breeds, their characteristics and endangered breeds.

Assignment of breeds to breeding directions:

**Group**	**Breed**
Merino	MFS, MLS, MLW
Meat	CHA, DOS, IDF, SKF, SUF, TEX, WKF
Country	BLS, BRI, COF, LES, RHO, RPL, WAD
Mountain/Stone	AST, BBS, KST, WBS
Heath	GGH, SKU, WGH, WHH
Milk	OMS
Exotic	BDC, KAM, NOL, OUS

Merino:

Merino is a breed with excellent wool quality. All Merinos have their origin in Spanish sheep, which were founded in the Middle ages. In Germany there are three different types of merinos. The German Merino (MLS) was created in the 19th century by crossing local breeds with fine wool merino sheep from Spain and South France. German mutton merino (MFS) has its origin in French merinos and English dual-purpose sheep breeds (fine wool and meat production). The German merino longwool (MLW) was bred in the former Democratic Republic of Germany by crossbreeding of German Merino with Corridale (USA, wool quality), British Lincoln (long wool and strong body conformation) and North Caucasian meat wool sheep.

All merinos in Germany are polled, medium sized with white wool, legs and face and breed out-of-season. Merinos are bred as dual-purpose breed for wool and meat quality.

The Merinos are hardy and robust sheep for economic lamb production under different production intensities. The chest is broad and sufficiently advanced. The metacarpus is long, with a tight, broad back, good rib curvature and long, deep flanks. The hindquarters show a long, broad pelvis and well fleshed inner and outer haunches. Merino ewes are fast-growing and show a high fattening performance and carcass quality with very good feed conversion. They are suitable for grazing, marching and horsemanship with best suitability for landscape maintenance in herd and paddock management.

Meat:

Meat sheep include breeds imported from British Islands, France, The Netherlands and South Africa. A few breeds originated in Germany through crossing local breeds with English meat breeds in the 19th century. Meat breeds produce both meat and wool, nearly reaching the quality of the fine merino wool. These breeds produce lambs with high quality carcass and reach high daily weight gains. Improvement of daily gain, muscle conformation and meat quality are important breeding objectives. The number of meat breeds is large.

Medium to large-framed, fast-growing animals with distinctive meat parts are desired. These include a deep, broad, forward chest, a deep, broad rump and a long back that is broad and well muscled. Also a broad, long pelvis with full outer and inner haunches that reach down low.

Country:

This group of sheep breeds is very heterogeneous and includes breeds which were not crossed with fine merino wool sheep nor with meat sheep breeds from foreign countries. Country sheep are

unpretentious and are therefore suitable for the maintenance of harsh and low-yielding landscapes. These breeds were locally developed and adapted to their ecosystems. The breeding programmes of these breeds focus primarily on breeding an undemanding, marchable and hardy sheep that impresses with a stable foundation and good claw health.

Mountain/Stone:

The mountain sheep breeds are mainly kept in the alpine and pre-alpine regions of South Germany in Bavaria and Swabia. Due to their long, coarse wool, they are particularly suitable for keeping in areas with high levels of rainfall. Many of these breeds are endangered due to decreasing numbers of breeding animals.

The group of mountain and stone sheep has in common the breeding of an early-maturing sheep with high fertility, longevity and best mothering qualities, which is adaptable to harsh high mountain conditions. The desired conformation of the stone sheep is a fine-boned, small to medium-sized sheep with a broad and deep body, while the mountain sheep are more of the medium to medium-large frame type.

Heath:

These breeds have their origin from heath- (German gray heath, German White heath) and moorlands (white polled heath) of North Germany and are suitable for poor soil. These sheep are managing the landscape through grazing heathland or moorland. Heath sheep are easy to maintain. The earliest reports date back to 18th century. Heath sheep are dual-coated with a small to moderate body size and a short tail.

Since the four heather breeds studied in this study are of particular regional importance in Germany, they were assigned to an extra group. These breeds are indispensable for the landscape management of the heath and are particularly characterised by their frugality and robustness. In former times they served as versatile meat and wool suppliers and today they improve the fertility of the heath soils, especially through their dung. Today the wool is no longer processed, it is too coarse. The meat yield of the animals is very low as they are frugal, light, small- to medium-framed land sheep.

Milk:

The East Friesian milk sheep originated from East Friesia and early reports go back to 16th century. East Friesian sheep are bred in three wool colour variants including white, black-brown and piebald. This breed is large sized with a coarse wool and low to moderate muscularity. The main breeding aim is improvement of milk production. Lacaune became popular in the last time and an increasing number of this French milk sheeps are entering the herdbook in Germany. Milk is mainly used for cheese production.

Exotic:

In this group, we subsumed a number of newly introduced breeds. This group contains hair sheep (KAM, NOL), meat sheep from France (BDC), and wool sheep (OUS). Quessant are short-tailed and one of the smallest sheep breeds. Their origin is from the coast of Brittany in France.

The group of exotic breeds also includes breeds that are kept and bred more as hobby animals and therefore do not correspond to a uniform breeding direction.

According to the Society for the Conservation of Old and Endangered Livestock Breeds (GEH), the following of the breeds studied in this work are among the endangered breeds:

Alpine Steinschaf
Bentheim
Brown Mountain
Carinthian
Dorper
Leine
East Friesian
German Grey Heath
German White Heath
Ile-de-France
Krainer Steinschaf
Merino Longwool
Nolana
Ouessant
Pomeranian Coarsewool
Skudde
Wald
White Mountain
White Polled Heath
Whiteheaded Mutton

The two breeds Coburger Fuchsschaf and Rhönschaf are on the pre-warning level.

Detailed information on sheep breeds in Germany can be found at: https://www.g-e-h.de/rassebeschreibungen/72-rassebeschreibungen-schafe
