# Supplementary material for: Effect of Individual Rate of Inbreeding, Recent and Ancestral Inbreeding on Wool Quality, Muscling Conformation and Exterior in German Sheep Breeds
Source: Animals (Basel). 2023 Oct 26;13(21):3329. doi: 10.3390/ani13213329 (PMC10648841; doi:10.3390/ani13213329)
Supplement: Supplementary file 1 [file animals-13-03329-s001.zip › Table S6a-6d.ANIMAL_Regression coefficients_Wool quality.pdf]

Table S6a. Animal model linear regression coefficients of the individual rate of inbreeding (ΔF_i) on the final score of wool quality, with their corresponding standard errors (SE) and *p*-Values by breed.

Breed	ΔF_i	SE	*p*-Value
AST	-0.9542	0.9360	0.1540
BBS	-0.3613	1.5272	0.4065
BDC	0.3481	1.6436	0.4161
BLS	-1.4458	2.6114	0.2899
BRI	-1.1097	1.5787	0.2411
CHA	-2.8162	1.2068	0.0098
COF	-0.5659	1.1380	0.3095
DOS	0.3796	3.9094	0.4613
GGH	-6.1883	1.7364	0.0002
IDF	-0.1552	0.6890	0.4109
KST	-1.1136	1.0829	0.1519
LES	-1.4700	1.4420	0.1540
MFS	-0.3379	1.4472	0.4077
MLS	1.3985	0.8942	0.0589
MLW	-4.1610	1.7332	0.0082
NOL	8.5836	3.3786	0.0055
OMS	-0.6542	1.6202	0.3432
OUS	0.0228	1.3981	0.4935
RHO	-1.5901	0.9693	0.0505
RPL	0.6745	2.9360	0.4091
SKF	-0.5115	1.0143	0.3070
SKU	-5.1689	1.8410	0.0025
SUF	0.2766	0.6154	0.3265
SWS	-0.2022	3.3148	0.4757
TEX	-0.9457	0.9532	0.1606
WAD	-1.2848	0.9255	0.0825
WBS	-2.0749	2.6102	0.2133
WGH	-2.9038	2.2408	0.0975
WHH	-0.7837	1.7124	0.3236
WKF	-3.2387	1.5241	0.0168

Table S6b. Animal model linear regression coefficients of the ancestral (F_{a_Kal}) and new (F_{a_New}) inbreeding coefficients according to Kalinowski on the final score of wool quality, with their corresponding standard errors (SE) and *p*-Values by breed.

Breed	F_{a_Kal}	SE	*p*-Value	F_{a_New}	SE	*p*-Value
AST	0.2899	1.0710	0.3933	-0.8715	0.5406	0.0535
BBS	-1.4034	1.4916	0.1734	0.0348	0.5250	0.4736
BDC	-19.1363	11.0505	0.0417	0.5022	0.7683	0.2567
BLS	0.0420	2.3290	0.4928	-0.3907	0.5255	0.2286
BRI	0.0873	3.1952	0.4891	-0.3255	0.7589	0.3340
CHA	5.1965	5.4995	0.1724	-2.2401	0.8207	0.0032
COF	0.7142	0.9401	0.2237	-0.3093	0.3343	0.1774
DOS	-1.1882	2.7172	0.3310	0.4099	1.5088	0.3929
GGH	-2.6005	1.8449	0.0793	-0.6608	0.4935	0.0903
IDF	-0.4818	2.0307	0.4062	-0.1765	0.3543	0.3092
KST	-1.3148	1.1342	0.1232	0.0316	0.4662	0.4730

**LES**	-2.5909	1.3530	0.0278	0.1733	0.4645	0.3545
**MFS**	-2.2667	2.0393	0.1332	0.1523	0.3922	0.3489
**MLS**	1.3132	0.8237	0.0554	-0.0380	0.2194	0.4312
**MLW**	2.6876	2.9251	0.1791	-1.2896	0.4978	0.0048
**NOL**	-1.0760	5.3414	0.4202	2.9988	1.6820	0.0373
**OMS**	1.9198	1.3552	0.0783	-0.4894	0.4471	0.1368
**OUS**	-0.2698	0.6932	0.3486	-0.0006	0.5353	0.4996
**RHO**	-0.7098	1.3374	0.2978	-0.4634	0.3346	0.0830
**RPL**	0.6541	0.9905	0.2545	0.1818	0.5455	0.3695
**SKF**	1.7192	1.3725	0.1052	-0.2830	0.2565	0.1349
**SKU**	2.3075	1.6618	0.0825	-2.2237	0.7035	0.0008
**SUF**	1.3660	1.8586	0.2312	0.0159	0.2308	0.4725
**SWS**	3.7350	20.7105	0.4284	-0.0633	1.4972	0.4831
**TEX**	0.1919	1.0295	0.4261	-0.1442	0.2445	0.2777
**WAD**	0.7183	0.5434	0.0931	-1.0882	0.4122	0.0041
**WBS**	1.7401	2.0650	0.1997	-0.9236	0.7405	0.1062
**WGH**	1.1483	1.6584	0.2443	-1.2351	0.8249	0.0672
**WHH**	2.1148	1.5071	0.0803	-0.8483	0.6059	0.0807
**WKF**	-0.8036	2.2098	0.3581	-0.5141	0.4345	0.1184

Table S6c. Animal model linear regression coefficients of the inbreeding coefficient (F) and interaction between F and the ancestral inbreeding coefficient according to Ballou ($F \times F_{a\_Bal}$) on the final score of wool quality, with their corresponding standard errors (SE) and *p*-Values by breed.

**Breed**	**F**	**SE**	***p*-Value**	**$F \times F_{a\_Bal}$**	**SE**	***p*-Value**
**AST**	-0.5194	0.3440	0.0655	2.2760	2.9180	0.2177
**BBS**	-0.2793	0.3545	0.2154	-0.5141	3.5888	0.4430
**BDC**	-0.1205	0.6767	0.4293	-39.7204	24.0558	0.0494
**BLS**	-0.2102	0.4447	0.3182	-3.7858	8.3315	0.3248
**BRI**	-0.2734	0.6054	0.3258	1.8893	7.3610	0.3987
**CHA**	-1.8440	0.7634	0.0079	18.8205	12.8791	0.0720
**COF**	-0.0836	0.2054	0.3420	0.5851	2.5243	0.4084
**DOS**	-0.0860	0.9164	0.4626	-8.4056	7.3203	0.1254
**GGH**	-0.9940	0.2947	0.0004	-3.2515	5.6376	0.2821
**IDF**	-0.1989	0.3136	0.2630	0.0698	5.0181	0.4945
**KST**	-0.2193	0.3197	0.2464	-2.7148	3.0361	0.1856
**LES**	-0.4202	0.2817	0.0679	-7.1241	2.8423	0.0061
**MFS**	-0.1397	0.2734	0.3047	-9.4990	5.0608	0.0303
**MLS**	0.1937	0.1391	0.0819	1.6827	2.2764	0.2299
**MLW**	-0.8294	0.3267	0.0056	11.3775	8.9318	0.1014
**NOL**	2.2276	1.2439	0.0367	-9.5945	15.9983	0.2743
**OMS**	0.0480	0.2518	0.4244	6.1773	3.3977	0.0345
**OUS**	-0.1026	0.2993	0.3659	-1.3056	1.7922	0.2332
**RHO**	-0.5037	0.2275	0.0134	-0.3335	3.3537	0.4604
**RPL**	0.1169	0.5276	0.4123	1.8512	2.4856	0.2282
**SKF**	-0.0291	0.1649	0.4300	5.8972	3.2613	0.0353
**SKU**	-1.0283	0.3864	0.0039	7.0981	4.4713	0.0562
**SUF**	0.0979	0.2005	0.3127	1.9870	3.9864	0.3091
**SWS**	0.0769	1.2205	0.4749	40.0136	48.5992	0.2052
**TEX**	-0.1906	0.2456	0.2189	2.5459	4.0843	0.2665
**WAD**	-0.3504	0.2330	0.0663	2.3614	1.2186	0.0263

**WBS**	-0.2686	0.4065	0.2544	3.4820	5.3191	0.2564
**WGH**	-0.5069	0.4104	0.1084	3.5026	4.5599	0.2212
**WHH**	-0.0586	0.2788	0.4168	4.1656	3.8051	0.1368
**WKF**	-0.5419	0.2727	0.0235	3.1349	5.0102	0.2658

Table S6d. Animal model linear regression coefficients of the inbreeding depression derived from the individual rate of inbreeding (ΔF_i), the ancestral (F_{a_Kal}) and new (F_{a_New}) inbreeding coefficient according to Kalinowski, inbreeding (F) and interaction between F and the ancestral inbreeding coefficient according to Ballou ($F \times F_{a\_Bal}$) on the final score of wool quality, with their corresponding standard deviations (SD), standard errors (SE) and the 95% confidence interval (95% CI), the 5% confidence interval (5% CI) and *p*-Values for all breeds and the six breeding directions (BD) of merino (MER), meat (MEA), country (CON), mountain (MON), heath (HEA) and exotic (EXO).

		**For all breeds**	**BD**					
			MER	MEA	CON	MON	HEA	EXO
**ΔF_i**	Mean	-0.9675	-1.0335	-0.9017	-0.9703	-1.2939	-3.7612	2.9848
	SD	2.4733	2.8443	1.3814	0.8008	0.5247	2.4134	4.8514
	SE	0.4515	1.6421	0.4884	0.3027	0.2623	1.2067	2.8009
	95% CI	1.3985	1.3985	0.3796	0.6745	-0.9542	-0.7837	8.5836
	5% CI	-5.1689	-4.1610	-3.2387	-1.5901	-2.0749	-6.1883	0.0228
	p-Value	0.0407	0.5934	0.1074	0.0185	0.0160	0.0526	0.3982
**F_{a_Kal}**	Mean	0.0630	0.5780	1.2169	-0.1550	1.7748	0.7425	-6.8273
	SD	4.1744	2.5577	2.2758	1.1910	3.3164	2.2857	10.6675
	SE	0.7621	1.4767	0.8046	0.4501	1.6582	1.1428	6.1589
	95% CI	5.1965	2.6876	5.1965	0.7183	6.3839	2.3075	-0.2698
	5% CI	-2.6005	-2.2667	-1.1882	-2.5909	-1.3148	-2.6005	-19.1363
	p-Value	0.9346	0.7332	0.1742	0.7424	0.3629	0.5622	0.3831
**F_{a_New}**	Mean	-0.3705	-0.3918	-0.3744	-0.3174	-0.6910	-1.2419	1.1668
	SD	0.9229	0.7834	0.7987	0.4303	0.4846	0.6968	1.6064
	SE	0.1685	0.4523	0.2824	0.1626	0.2423	0.3484	0.9274
	95% CI	0.5022	0.1523	0.4099	0.1818	0.0316	-0.6608	2.9988
	5% CI	-2.2237	-1.2896	-2.2401	-1.0882	-1.000	-2.2237	-0.0006
	p-Value	0.0360	0.4777	0.2265	0.0988	0.0650	0.0377	0.3353
**F**	Mean	-0.2454	-0.2585	-0.3395	-0.2464	-0.4028	-0.6469	0.6682
	SD	0.6290	0.5218	0.6405	0.2113	0.1877	0.4588	1.3505
	SE	0.1148	0.3012	0.2264	0.0799	0.0939	0.2294	0.7797
	95% CI	0.1937	0.1937	0.0979	0.1169	-0.2193	-0.0586	2.2276
	5% CI	-1.0283	-0.8294	-1.8440	-0.5037	-0.6039	-1.0283	-0.1205
	p-Value	0.0412	0.4813	0.1775	0.0215	0.0233	0.0667	0.4818
**$F \times F_{a\_Bal}$**	Mean	1.5953	1.1871	8.0079	-0.6509	4.4298	2.8787	-16.8735
	SD	12.2412	10.4471	14.9665	3.5389	7.3386	4.3752	20.2154
	SE	2.2349	6.0316	5.2915	1.3376	3.6693	2.1876	11.6713
	95% CI	18.8205	11.3775	40.0136	2.3614	14.6759	7.0981	-1.3056
	5% CI	-9.5945	-9.4990	-8.4056	-7.1241	-2.7148	-3.2515	-39.7204
	p-Value	0.4811	0.8622	0.1740	0.6438	0.3138	0.2797	0.2851

Abbreviations for breeding directions: country: CON, exotic: EXO, heath: HEA, meat: MEA, merino: MER, mountain-stone: MON.
