# Supplementary material for: Effect of Individual Rate of Inbreeding, Recent and Ancestral Inbreeding on Wool Quality, Muscling Conformation and Exterior in German Sheep Breeds
Source: Animals (Basel). 2023 Oct 26;13(21):3329. doi: 10.3390/ani13213329 (PMC10648841; doi:10.3390/ani13213329)
Supplement: Supplementary file 1 [file animals-13-03329-s001.zip › Table S7a-7d.ANIMAL_Regression coefficients_Muscling conformation.pdf]

Table S7a. Animal model linear regression coefficients of the individual rate of inbreeding (ΔF_i) on the final score of muscling conformation, with their corresponding standard errors (SE) and *p*-Values by breed.

Breed	ΔF_i	SE	*p*-Value
AST	-1.0911	0.8438	0.0980
BBS	-1.2054	1.1869	0.1549
BDC	-0.9820	1.4740	0.2526
BLS	-2.5976	2.1559	0.1141
BRI	-2.2083	1.2892	0.0434
CHA	-1.6934	1.1982	0.0788
COF	-2.7473	0.9337	0.0016
DOS	-0.1846	3.1494	0.4766
GGH	-3.6689	1.6053	0.0111
IDF	-1.6628	0.5881	0.0023
KST	-0.7863	0.9823	0.2117
LES	-5.1050	1.3059	0.0000
MFS	-3.7561	1.2061	0.0009
MLS	-1.8018	0.6918	0.0046
MLW	-4.1333	1.6199	0.0054
NOL	2.3645	1.9722	0.1153
OMS	-1.8869	1.4722	0.1000
OUS	-1.3754	1.1307	0.1119
RHO	-1.9494	0.9136	0.0164
RPL	-0.8015	2.0669	0.3491
SKF	-1.9682	0.8962	0.0140
SKU	-3.8410	1.4076	0.0032
SUF	-0.6450	0.5339	0.1135
SWS	-3.2524	3.0458	0.1428
TEX	-1.6722	0.8611	0.0261
WAD	-0.8194	0.8429	0.1655
WBS	-3.5339	2.0570	0.0429
WGH	-0.6613	1.7649	0.3539
WHH	-2.9319	1.3511	0.0150
WKF	-3.2653	1.4738	0.0134

Table S7b. Animal model linear regression coefficients of the ancestral (F_{a_Kal}) and new (F_{a_New}) inbreeding coefficient according to Kalinowski on the final score of muscling conformation, with their corresponding standard errors (SE) and *p*-Values by breed.

Breed	F_{a_Kal}	SE	*p*-Value	F_{a_New}	SE	*p*-Value
AST	-1.1606	0.9839	0.1191	-0.5487	0.4869	0.1299
BBS	-0.6767	1.1547	0.2789	-0.2668	0.4086	0.2569
BDC	-10.1053	9.9432	0.1547	-0.2021	0.6877	0.3844
BLS	-1.7203	1.9227	0.1855	-0.1981	0.4339	0.3240
BRI	-0.1539	2.5205	0.4757	-0.7596	0.6200	0.1103
CHA	-4.3568	5.3715	0.2087	-0.9245	0.8151	0.1284
COF	-1.0711	0.7709	0.0824	-0.4410	0.2743	0.0539
DOS	-1.1207	2.0736	0.2944	-0.1995	1.2203	0.4351
GGH	-2.0678	1.7231	0.1151	-0.3538	0.4553	0.2186
IDF	0.6086	1.7533	0.3643	-0.8966	0.3043	0.0016
KST	-0.0019	1.0155	0.4993	-0.0958	0.4234	0.4105

**LES**	-1.9602	1.2165	0.0536	-0.7782	0.4201	0.0320
**MFS**	-1.6280	1.7054	0.1699	-0.6623	0.3262	0.0212
**MLS**	-0.7098	0.6307	0.1302	-0.2261	0.1696	0.0912
**MLW**	1.5615	2.7379	0.2842	-1.2333	0.4651	0.0040
**NOL**	-2.2495	3.0152	0.2278	1.1077	0.9834	0.1300
**OMS**	-0.4612	1.2295	0.3538	-0.2866	0.4061	0.2402
**OUS**	-0.7219	0.6539	0.1348	-0.0432	0.4402	0.4609
**RHO**	0.9663	1.2602	0.2216	-0.7661	0.3137	0.0073
**RPL**	0.0699	0.6713	0.4585	-0.6095	0.3883	0.0582
**SKF**	1.0049	1.2173	0.2045	-0.5550	0.2267	0.0072
**SKU**	-1.7504	1.2741	0.0847	-0.5645	0.5356	0.1460
**SUF**	-2.0190	1.6257	0.1071	-0.2471	0.2000	0.1083
**SWS**	-1.7946	19.4073	0.4632	-1.2420	1.3913	0.1860
**TEX**	-2.3810	0.9295	0.0052	-0.2394	0.2208	0.1391
**WAD**	0.1287	0.5192	0.4021	-0.4664	0.3753	0.1070
**WBS**	-1.3318	1.6160	0.2049	-0.3025	0.5857	0.3028
**WGH**	-1.5163	1.2839	0.1188	0.3893	0.6495	0.2745
**WHH**	2.3371	1.1746	0.0233	-1.3846	0.4768	0.0018
**WKF**	-2.7020	2.1085	0.1000	-0.2125	0.4152	0.3044

Table S7c. Animal model linear regression coefficients of the inbreeding coefficient (F) and interaction between F and the ancestral inbreeding coefficient according to Ballou ($F \times F_{a\_Bal}$) on the final score of muscling conformation, with their corresponding standard errors (SE) and *p*-Values by breed.

**Breed**	**F**	**SE**	***p*-Value**	**$F \times F_{a\_Bal}$**	**SE**	***p*-Value**
**AST**	-0.7251	0.3113	0.0099	-4.2242	2.6430	0.0550
**BBS**	-0.3563	0.2752	0.0977	-0.0358	2.7883	0.4949
**BDC**	-0.5112	0.6075	0.2000	-34.2169	21.6796	0.0572
**BLS**	-0.4497	0.3671	0.1103	-3.4008	6.8783	0.3105
**BRI**	-0.6834	0.4917	0.0823	4.5515	5.8650	0.2189
**CHA**	-1.0735	0.7566	0.0780	-6.3671	12.6464	0.3073
**COF**	-0.5772	0.1685	0.0003	-2.3068	2.0710	0.1327
**DOS**	-0.5557	0.7328	0.2241	0.9225	5.8003	0.4368
**GGH**	-0.6416	0.2732	0.0094	-6.4012	5.2058	0.1094
**IDF**	-0.7831	0.2680	0.0017	4.7613	4.3350	0.1360
**KST**	-0.0794	0.2884	0.3915	0.9243	2.7464	0.3682
**LES**	-1.0414	0.2548	0.0000	-3.4042	2.5659	0.0923
**MFS**	-0.7854	0.2278	0.0003	-4.2960	4.2085	0.1537
**MLS**	-0.3155	0.1075	0.0017	-0.7142	1.7505	0.3416
**MLW**	-0.9066	0.3054	0.0015	6.3911	8.3511	0.2220
**NOL**	0.4828	0.7185	0.2508	-13.6210	9.0989	0.0672
**OMS**	-0.3348	0.2287	0.0716	2.0264	3.0846	0.2556
**OUS**	-0.2941	0.2452	0.1152	-0.9278	1.6095	0.2822
**RHO**	-0.4981	0.2138	0.0099	4.1284	3.1529	0.0952
**RPL**	-0.1432	0.3694	0.3491	1.0391	1.6993	0.2704
**SKF**	-0.3589	0.1457	0.0069	-0.3512	2.8876	0.4516
**SKU**	-0.8329	0.2956	0.0024	0.0553	3.4207	0.4936
**SUF**	-0.3412	0.1741	0.0250	-3.9221	3.4791	0.1298
**SWS**	-1.2526	1.1183	0.1313	-35.4659	45.3597	0.2171
**TEX**	-0.5379	0.2217	0.0076	-6.0769	3.6893	0.0498
**WAD**	-0.2336	0.2136	0.1371	1.1205	1.1513	0.1652

**WBS**	-0.5240	0.3198	0.0507	-1.8721	4.1970	0.3278
**WGH**	-0.2158	0.3221	0.2514	-4.8711	3.5777	0.0867
**WHH**	-0.3836	0.2197	0.0404	5.2029	2.9985	0.0414
**WKF**	-0.5331	0.2638	0.0216	-7.1425	4.8435	0.0702

Table S7d. Animal model linear regression coefficients of the inbreeding depression derived from the individual rate of inbreeding (ΔF_i), the ancestral (F_{a_Kal}) and new (F_{a_New}) inbreeding coefficient according to Kalinowski, inbreeding (F) and interaction between F and the ancestral inbreeding coefficient according to Ballou ($F \times F_{a\_Bal}$) on the final score of muscling conformation, with their corresponding standard deviations (SD), standard errors (SE) and the 95 % Confidence interval (95 % CI), the 5 % confidence interval (5 % CI) and *p*-Values for all breeds and the six breeding directions (BD) of merino (MER), meat (MEA), country (CON), mountain (MON), heath (HEA) and exotic (EXO).

		**For all breeds**	**BD**					
			MER	MEA	CON	MON	HEA	EXO
**ΔF_i**	mean	-1.9616	-3.2304	-1.7930	-2.3184	-1.4007	-2.7758	0.0024
	SD	1.5191	1.2515	1.0872	1.4562	1.4704	1.4638	2.0551
	SE	0.2773	0.7226	0.3844	0.5504	0.7352	0.7319	1.1865
	95 % CI	-0.18460	-1.8018	-0.1846	-0.8015	-0.1915	-0.6613	2.3645
	5 % CI	-4.1333	-4.1333	-3.2653	-5.1050	-3.5339	-3.8410	-1.3754
	p-Value	<.0001	0.0466	0.0023	0.0056	0.1528	0.0322	0.9986
**F_{a_Kal}**	mean	-1.2755	-0.2588	-1.5951	-0.5344	-1.1131	-0.7494	-4.3589
	SD	2.1780	1.6419	1.7538	1.0746	0.8163	2.0700	5.0348
	SE	0.3976	0.9480	0.6200	0.4062	0.4082	1.0350	2.9068
	95 % CI	1.5615	1.5615	1.0049	0.9663	-0.0019	2.3371	-0.7219
	5 % CI	-4.3568	-1.6280	-4.3568	-1.9602	-1.9582	-2.0678	-10.1053
	p-Value	0.0033	0.8105	0.0369	0.2363	0.0721	0.5214	0.2725
**F_{a_New}**	mean	-0.4444	-0.7072	-0.5646	-0.5741	-0.3343	-0.4784	0.2875
	SD	0.4846	0.5051	0.4075	0.2179	0.1889	0.7296	0.7148
	SE	0.0884	0.2916	0.1441	0.0824	0.0944	0.3648	0.4127
	95 % CI	0.3893	-0.2261	-0.1995	-0.1981	-0.0958	0.3893	1.1077
	5 % CI	-1.2420	-1.2333	-1.2420	-0.7782	-0.5487	-1.3846	-0.2021
	p-Value	<.0001	0.1361	0.0058	0.0004	0.0383	0.2811	0.5581
**F**	mean	-0.5185	-0.6692	-0.6795	-0.5181	-0.4385	-0.5185	-0.10750
	SD	0.3386	0.3122	0.3313	0.2976	0.2699	0.2732	0.5226
	SE	0.0618	0.1803	0.1171	0.1125	0.1350	0.1366	0.3017
	95 % CI	-0.0794	-0.3155	-0.3412	-0.1432	-0.0794	-0.2158	0.4828
	5 % CI	-1.0735	-0.9066	-1.2526	-1.0414	-0.7251	-0.8329	-0.5112
	p-Value	<.0001	0.0655	0.0007	0.0037	0.0475	0.0321	0.7557
**$F \times F_{a\_Bal}$**	mean	-3.7863	0.4603	-6.7052	0.2468	-2.5759	-1.5035	-16.2552
	SD	9.5145	5.4395	12.3384	3.3701	2.7077	5.2515	16.8001
	SE	1.7371	3.1405	4.3623	1.2738	1.3539	2.6257	9.6996
	95 % CI	5.2029	6.3911	4.7613	4.5515	0.9243	5.2029	-0.9278
	5 % CI	-34.2169	-4.2960	-35.4659	-3.4042	-5.1316	-6.4012	-34.2169
	p-Value	0.0375	0.8969	0.1682	0.8528	0.1532	0.6070	0.2358

Abbreviations for breeding directions: country: CON, exotic: EXO, heath: HEA, meat: MEA, merino: MER, mountain-stone: MON.
