# Supplementary material for: Effect of Individual Rate of Inbreeding, Recent and Ancestral Inbreeding on Wool Quality, Muscling Conformation and Exterior in German Sheep Breeds
Source: Animals (Basel). 2023 Oct 26;13(21):3329. doi: 10.3390/ani13213329 (PMC10648841; doi:10.3390/ani13213329)
Supplement: Supplementary file 1 [file animals-13-03329-s001.zip › Table S8a-8d.ANIMAL_Regression coefficients_Exterior.pdf]

Table S8a. Animal model linear regression coefficients of the individual rate of inbreeding (ΔF_i) on the final score of exterior, with their corresponding standard errors (SE) and *p*-V*alues* by breed.

Breed	ΔF_i	SE	*p*-Value
AST	-1.0067	0.8799	0.1263
BBS	-0.1776	1.4015	0.4496
BDC	0.0293	1.8337	0.4936
BLS	0.3330	2.4615	0.4462
BRI	-2.7081	1.4802	0.0337
CHA	-0.8124	1.3850	0.2787
COF	-2.7352	1.0294	0.0039
DOS	-4.1725	3.1952	0.0958
GGH	-1.9643	1.6564	0.1178
IDF	-1.8614	0.6411	0.0018
KST	-0.3886	1.2011	0.3731
LES	-3.7127	1.3997	0.0040
MFS	-1.7780	1.3366	0.0917
MLS	-1.8192	0.7582	0.0082
MLW	-2.9447	1.5791	0.0311
NOL	3.6668	2.4338	0.0660
OMS	-3.0262	1.6284	0.0316
OUS	-0.5772	1.6471	0.3630
RHO	-1.1975	0.9808	0.1111
RPL	0.9515	2.5858	0.3564
SKF	-2.3858	1.0148	0.0094
SKU	-2.8323	1.8814	0.0661
SUF	-1.7160	0.6468	0.0040
SWS	-10.7442	3.8181	0.0024
TEX	-1.5534	1.0646	0.0723
WAD	-2.6114	0.9247	0.0024
WBS	-1.5095	2.1923	0.2456
WGH	-2.7329	1.8753	0.0725
WHH	-1.7367	1.4430	0.1144
WKF	-6.8340	1.7594	0.0001

Table S8b. Animal model linear regression coefficients of the ancestral (F_{aKal}) and new (F_{aNew}) inbreeding coefficient according to Kalinowski on the final score of exterior, with their corresponding standard errors (SE) and *p*-V*alues* by breed.

Breed	F_{a_Kal}	SE	*p*-Value	F_{a_New}	SE	*p*-Value
AST	-0.330	0.983	0.369	-0.7219	0.5085	0.0779
BBS	-0.229	1.366	0.434	-0.0024	0.4819	0.4980
BDC	1.544	12.515	0.451	0.0729	0.8539	0.4660
BLS	-0.383	2.195	0.431	0.0142	0.4954	0.4886
BRI	0.023	2.866	0.497	-0.9730	0.7118	0.0858
CHA	4.207	6.271	0.251	-1.1781	0.9422	0.1056
COF	-0.500	0.844	0.277	-0.5826	0.3028	0.0272
DOS	1.224	2.042	0.274	-2.1012	1.2494	0.0463
GGH	-3.487	1.768	0.024	0.2374	0.4704	0.3069
IDF	0.173	1.901	0.464	-0.9179	0.3308	0.0028
KST	-0.372	1.232	0.381	0.1658	0.5183	0.3745

**LES**	-0.657	1.297	0.306	-0.6963	0.4498	0.0608
**MFS**	2.317	1.859	0.106	-0.7162	0.3631	0.0243
**MLS**	-0.805	0.674	0.116	-0.2133	0.1854	0.1250
**MLW**	4.430	2.665	0.048	-1.3081	0.4536	0.0020
**NOL**	3.438	3.679	0.175	0.5194	1.2139	0.3344
**OMS**	1.891	1.337	0.079	-1.0590	0.4487	0.0091
**OUS**	-1.204	0.887	0.087	0.3830	0.6347	0.2731
**RHO**	1.101	1.342	0.206	-0.6131	0.3386	0.0351
**RPL**	-0.033	0.842	0.484	0.1656	0.4852	0.3664
**SKF**	0.781	1.366	0.284	-0.5944	0.2565	0.0102
**SKU**	0.022	1.704	0.495	-0.5049	0.7152	0.2401
**SUF**	0.973	1.968	0.310	-0.6855	0.2427	0.0024
**SWS**	-2.850	24.581	0.454	-3.9520	1.7573	0.0123
**TEX**	-2.442	1.141	0.016	-0.1917	0.2725	0.2409
**WAD**	-0.100	0.595	0.434	-1.0150	0.4114	0.0068
**WBS**	0.390	1.695	0.409	-0.3455	0.6288	0.2913
**WGH**	0.466	1.307	0.361	-0.8640	0.6891	0.1050
**WHH**	0.722	1.212	0.276	-0.6454	0.5048	0.1005
**WKF**	0.572	2.476	0.409	-1.4900	0.4898	0.0012

Table S8c. Animal model linear regression coefficients of the inbreeding coefficient (F) and interaction between F and the ancestral inbreeding coefficient according to Ballou ($F \times F_{a\_Bal}$) on the final score of exterior, with their corresponding standard errors (SE) and *p*-V*alues* by breed.

**Breed**	**F**	**SE**	***p*-Value**	**$F \times F_{a\_Bal}$**	**SE**	***p*-Value**
**AST**	-0.5817	0.3212	0.0351	-2.4079	2.7298	0.1889
**BBS**	-0.0517	0.3251	0.4368	1.4912	3.2922	0.3253
**BDC**	0.0982	0.7576	0.4484	6.1622	27.4104	0.4111
**BLS**	0.0491	0.4192	0.4534	-3.6639	7.8532	0.3204
**BRI**	-0.8337	0.5638	0.0696	4.6328	6.6869	0.2442
**CHA**	-0.8890	0.8757	0.1550	14.8244	14.7041	0.1567
**COF**	-0.5687	0.1855	0.0011	-1.5402	2.2789	0.2496
**DOS**	-0.9189	0.7403	0.1073	8.4086	5.8671	0.0759
**GGH**	-0.4179	0.2815	0.0688	-11.9653	5.3753	0.0130
**IDF**	-0.8349	0.2919	0.0021	2.9009	4.7024	0.2687
**KST**	0.0468	0.3514	0.4470	1.8756	3.3508	0.2878
**LES**	-0.6918	0.2730	0.0056	-0.4942	2.7452	0.4286
**MFS**	-0.3266	0.2521	0.0976	5.1465	4.6529	0.1343
**MLS**	-0.3189	0.1176	0.0033	-1.3934	1.8920	0.2307
**MLW**	-0.6430	0.2976	0.0154	13.3815	8.1380	0.0501
**NOL**	1.0605	0.8835	0.1150	4.1859	11.1273	0.3534
**OMS**	-0.4050	0.2527	0.0545	6.3389	3.3749	0.0302
**OUS**	-0.2379	0.3549	0.2513	-3.3137	2.2284	0.0685
**RHO**	-0.3485	0.2301	0.0649	4.0465	3.3836	0.1159
**RPL**	0.0657	0.4624	0.4435	0.7328	2.1317	0.3655
**SKF**	-0.4169	0.1649	0.0057	3.0121	3.2535	0.1773
**SKU**	-0.3694	0.3951	0.1749	3.9287	4.5718	0.1951
**SUF**	-0.5703	0.2110	0.0034	2.0843	4.2124	0.3104
**SWS**	-3.9145	1.4007	0.0026	36.7491	57.2243	0.2604
**TEX**	-0.5441	0.2734	0.0233	-7.6156	4.5543	0.0472
**WAD**	-0.6630	0.2359	0.0025	1.0716	1.3069	0.2061

**WBS**	-0.1629	0.3393	0.3156	-0.1800	4.4867	0.4840
**WGH**	-0.4228	0.3392	0.1063	3.8311	3.7658	0.1545
**WHH**	-0.2852	0.2337	0.1112	-0.1753	3.1858	0.4781
**WKF**	-1.2071	0.3147	0.0001	0.7957	5.7779	0.4452

Table S8d. Animal model linear regression coefficients of the inbreeding depression derived from the individual rate of inbreeding (ΔF_i), the ancestral (F_{a_Kal}) and new (F_{a_New}) inbreeding coefficient according to Kalinowski, inbreeding (F) and interaction between F and the ancestral inbreeding coefficient according to Ballou ($F \times F_{a\_Bal}$) on the final score of exterior, with their corresponding standard deviations (SD), standard errors (SE) and the 95% Confidence interval (95% CI), the 5 % confidence interval (5% CI) and *p*-Values for all breeds and the six breeding directions (BD) of merino (MER), meat (MEA), country (CON), mountain (MON), heath (HEA) and exotic (EXO).

		**For all breeds**	**BD**					
			MER	MEA	CON	MON	HEA	EXO
**ΔF_i**	mean	-2.0354	-2.1806	-3.7600	-1.6686	-0.8968	-2.3166	1.0396
	SD	2.4400	0.6620	3.4148	1.7502	0.4802	0.5476	2.2953
	SE	0.4455	0.3822	1.2073	0.6615	0.2401	0.2738	1.3252
	95 % CI	0.9515	-1.7780	-0.8124	0.9515	-0.3886	-1.7367	3.6668
	5 % CI	-6.8340	-2.9447	-10.7442	-3.7127	-1.5095	-2.8323	-0.5772
	p-Value	<.0001	0.0294	0.0170	0.0451	0.0335	0.0035	0.5149
**F_{a_Kal}**	mean	0.3279	1.9807	0.3298	-0.0786	-0.3969	-0.5692	1.2596
	SD	1.7987	2.6335	2.2147	0.5786	0.6825	1.9666	2.3341
	SE	0.3284	1.5205	0.7830	0.2187	0.3412	0.9833	1.3476
	95 % CI	4.2072	4.4299	4.2072	1.1007	0.3902	0.7218	3.4383
	5 % CI	-2.8503	-0.8048	-2.8503	-0.6569	-1.2759	-3.4870	-1.2037
	p-Value	0.3264	0.3225	0.6862	0.7316	0.3289	0.6033	0.4486
**F_{a_New}**	mean	-0.6834	-0.7459	-1.3889	-0.5286	-0.3985	-0.4442	0.3251
	SD	0.8504	0.5480	1.1900	0.4562	0.4132	0.4778	0.2288
	SE	0.1553	0.3164	0.4207	0.1724	0.2066	0.2389	0.1321
	95 % CI	0.3830	-0.2133	-0.1917	0.1656	0.1658	0.2374	0.5194
	5 % CI	-2.1012	-1.3081	-3.9520	-1.0150	-0.7219	-0.8640	0.0729
	p-Value	0.0001	0.1425	0.0131	0.0221	0.1493	0.1599	0.1330
**F**	mean	-0.5325	-0.4295	-1.1620	-0.4273	-0.3550	-0.3738	0.3069
	SD	0.7634	0.1849	1.1406	0.3621	0.3580	0.0638	0.6739
	SE	0.1394	0.1068	0.4033	0.1369	0.1790	0.0319	0.3891
	95 % CI	0.0982	-0.3189	-0.4169	0.0657	0.0468	-0.2852	1.0605
	5 % CI	-1.2071	-0.6430	-3.9145	-0.8337	-0.7222	-0.4228	-0.2379
	p-Value	0.0007	0.0566	0.0236	0.0205	0.1416	0.0013	0.5128
**$F \times F_{a\_Bal}$**	mean	2.7898	5.71153	7.6449	0.6836	-2.0949	-1.0952	2.3448
	SD	8.5078	7.4036	13.3837	2.9522	4.1061	7.4947	4.9990
	SE	1.5533	4.2745	4.7319	1.1158	2.0531	3.7474	2.8862
	95 % CI	14.8244	13.3815	36.7491	4.6328	1.8756	3.9287	6.1622
	5 % CI	-7.6672	-1.3934	-7.6156	-3.6639	-7.6672	-11.9653	-3.3137
	p-Value	0.0829	0.3132	0.1502	0.5626	0.3827	0.7891	0.5019

Abbreviations for breeding directions: country: CON, exotic: EXO, heath: HEA, meat: MEA, merino: MER, mountain-stone: MON.
